# (*E*)-1-[(2-Fluoro­phen­yl)imino­meth­yl]-2-naphthol–(*Z*)-1-[(2-fluoro­phen­yl)amino­methyl­idene]naphthalen-2(1*H*)-one (0.57/0.43)

**DOI:** 10.1107/S1600536810014777

**Published:** 2010-04-28

**Authors:** Gökhan Alpaslan, Mustafa Macit, Orhan Büyükgüngör, Ahmet Erdönmez

**Affiliations:** a Department of Physics, Faculty of Arts & Science, Ondokuz Mayıs University, TR-55139 Kurupelit-Samsun, Turkey; b Department of Chemistry, Faculty of Arts & Science, Ondokuz Mayıs University, TR-55139 Kurupelit-Samsun, Turkey

## Abstract

The title Schiff base compound, 0.57C_17_H_12_FNO·0.43C_17_H_12_FNO, reveals both the enol (OH) and keto (NH) tautomeric forms with occupancies of 0.57 (6) and 0.43 (6), respectively. The tautomeric forms are stabilized by intra­molecular O—H⋯N (enol) and N—H⋯O (keto) hydrogen bonds. The dihedral angle between the naphthalene ring system and the benzene ring is 32.76 (1)°.

## Related literature

For the biological properties of Schiff bases, see: Lozier *et al.* (1975 ▶). For the coordination chemistry of Schiff bases, see: Kargar *et al.* (2009 ▶); Yeap *et al.* (2009 ▶). For Schiff base tautomerism, see: Hökelek *et al.* (2000 ▶); Kaitner & Pavlovic (1996 ▶); Karabıyık *et al.* (2007 ▶); Nazır *et al.* (2000 ▶); Odabaşoğlu *et al.* (2005 ▶); Yıldız *et al.* (1998 ▶); Tanak *et al.* (2009 ▶). For bond-length data, see: Allen *et al.* (1987 ▶). 
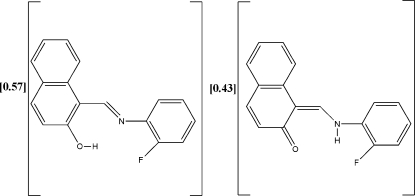


## Experimental

### 

#### Crystal data

0.57C_17_H_12_FNO·0.43C_17_H_12_FNO
*M* *_r_* = 265.28
Orthorhombic, 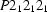

*a* = 7.2841 (3) Å
*b* = 12.2158 (6) Å
*c* = 14.5731 (7) Å
*V* = 1296.73 (10) Å^3^
*Z* = 4
Mo *K*α radiation
μ = 0.10 mm^−1^
*T* = 296 K
0.73 × 0.31 × 0.10 mm

#### Data collection

Stoe IPDSII diffractometer
Absorption correction: integration (*X-RED32*; Stoe & Cie, 2002 ▶) *T*_min_ = 0.967, *T*_max_ = 0.993
5655 measured reflections
1477 independent reflections
951 reflections with *I* > 2σ(*I*)
*R*_int_ = 0.040

#### Refinement

*R*[*F* ^2^ > 2σ(*F* ^2^)] = 0.047
*wR*(*F* ^2^) = 0.113
*S* = 0.97
1477 reflections
183 parameters
1 restraint
H-atom parameters constrained
Δρ_max_ = 0.23 e Å^−3^
Δρ_min_ = −0.14 e Å^−3^

### 

Data collection: *X-AREA* (Stoe & Cie, 2002 ▶); cell refinement: *X-AREA*; data reduction: *X-RED32* (Stoe & Cie, 2002 ▶); program(s) used to solve structure: *SHELXS97* (Sheldrick, 2008 ▶); program(s) used to refine structure: *SHELXL97* (Sheldrick, 2008 ▶); molecular graphics: *ORTEP-3 for Windows* (Farrugia, 1997 ▶); software used to prepare material for publication: *WinGX* (Farrugia, 1999 ▶).

## Supplementary Material

Crystal structure: contains datablocks I, global. DOI: 10.1107/S1600536810014777/ci5080sup1.cif

Structure factors: contains datablocks I. DOI: 10.1107/S1600536810014777/ci5080Isup2.hkl

Additional supplementary materials:  crystallographic information; 3D view; checkCIF report

## Figures and Tables

**Table 1:** Hydrogen-bond geometry (Å, °)

*D*—H⋯*A*	*D*—H	H⋯*A*	*D*⋯*A*	*D*—H⋯*A*
O1—H1*A*⋯N1	0.82	1.82	2.535 (4)	144
N1—H1*B*⋯O1	0.86	1.86	2.535 (4)	134

**Fig. 1.**
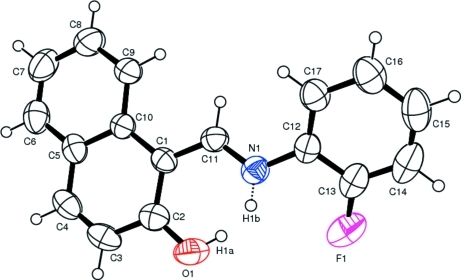
The molecular structure of (I), showing the atom-numbering scheme. Displacement ellipsoids are drawn at the 40% probability level. Both OH (solid O–H bond) and NH (dashed N–H bond) tautomers are shown.

## supplementary crystallographic information

### Comment

Schiff bases often exhibit various biological activities and in many cases were shown to have antibacterial, anticancer, anti-inflammatory and antitoxic properties (Lozier *et al.*, 1975). Schiff bases have also been used as versatile ligands in coordination chemistry (Kargar *et al.*, 2009; Yeap *et al.*, 2009). There are two types of intramolecular hydrogen bonds in Schiff bases, namely N—H···O in keto (NH) (Hökelek *et al.*, 2000) and N···H—O in enol (OH) (Odabaşoǧlu *et al.*, 2005) tautomeric forms. In the solid state, while OH tautomeric forms of Schiff bases are predominant in salicylaldimines (Kaitner & Pavlovic, 1996; Yıldız *et al.*,1998), both NH and OH forms have been found in naphthaldimine Schiff base compounds (Nazır *et al.*, 2000; Karabıyık *et al.*, 2007). Our investigations shows that in the title compound both OH (enol) and NH (keto) tautomers coexist with occupancies of 0.57 (6) and 0.43 (6), respectively. This evidence is also supported by the observed IR vibrational bands given in the experimental section.

An ORTEP-3 (Farrugia, 1997) plot of the molecule of (I) is shown in Fig.1. The C2—O1 [1.314 (5) Å] and C11—N1 [1.306 (4) Å] bond lengths are intermediate between the single and double C—O (1.362 and 1.222 Å, respectively) and C—N bond lengths (1.339 and 1.279 Å, respectively) (Allen *et al.*, 1987). Similar results were observed for 2-[(2,4-dimethylphenyl)iminomethyl]-3,5-dimethoxyphenol (Tanak *et al.*, 2009). The molecule of the title compound is not planar, with a dihedral angle of 32.76 (1)° between naphthalene and benzene rings. The molecular structure is stabilized by O—H···N or N—H···O hydrogen bonds.

### Experimental

The title compound was prepared by refluxing a mixture of a solution containing 2-hydroxy-1-naphthaldehyde (172 mg, 1 mmol) in 50 ml ethanol and a solution containing 2-fluoroaniline (111 mg, 1 mmol) in 30 ml ethanol. The reaction mixture was stirred for 3 h under reflux. The crystals of the title compound were obtained by slow evaporation of ethanol (yield 68%; m.p. 361-363 K). The FT-IR spectra of the title compound was recorded on a KBr pellets with a Schmadzu FT-IR 8900 spectrophotometer. IR (KBr) ν = 3558 (O–H), 3420 (N–H), 1698 (C═O) weak, 1620 (C═N) cm^-1^.

### Refinement

The absolute configuration could not be determined from X-ray data, as no strong anomalous scatterer is present; 1058 Friedel pairs were merged before the final refinement. All H atoms (except H1A and H1B) were placed in calculated positions and constrained to ride on their parent atoms, with C–H = 0.93 Å and *U*_iso_(H) = 1.2 *U*_eq_(C). Inspection of the tautomeric hydrogen atom's (H1) location in difference Fourier map between O1 and N1 indicated that there is a positional disorder for this H atom. Hence, atoms H1A and H1B bonded with O1 and N1 were positioned geometrically and their occupancies were refined to 0.57 (6) and 0.43 (6), respectively. A restraint (DELU instruction in SHELXL97, Sheldrick, 2008) was used in order to maintain a reasonable geometry and atomic displacement parameters for atoms C13 and F1.

### Crystal data

**Table:**

0.57C_17_H_12_FNO·0.43C_17_H_12_FNO	*F*(000) = 552
*M**_r_* = 265.28	*D*_x_ = 1.359 Mg m^−^^3^
Orthorhombic, *P*2_1_2_1_2_1_	Mo *K*α radiation, λ = 0.71073 Å
Hall symbol: P 2ac 2ab	Cell parameters from 4776 reflections
*a* = 7.2841 (3) Å	θ = 1.4–27.8°
*b* = 12.2158 (6) Å	µ = 0.10 mm^−^^1^
*c* = 14.5731 (7) Å	*T* = 296 K
*V* = 1296.73 (10) Å^3^	Needle, yellow
*Z* = 4	0.73 × 0.31 × 0.10 mm

### Data collection

**Table:**

Stoe IPDSII diffractometer	1477 independent reflections
Radiation source: fine-focus sealed tube	951 reflections with *I* > 2σ(*I*)
graphite	*R*_int_ = 0.040
Detector resolution: 6.67 pixels mm^-1^	θ_max_ = 26.0°, θ_min_ = 2.2°
ω scans	*h* = −8→8
Absorption correction: integration (*X-RED32*; Stoe & Cie, 2002)	*k* = −13→15
*T*_min_ = 0.967, *T*_max_ = 0.993	*l* = −17→16
5655 measured reflections	

### Refinement

**Table:**

Refinement on *F*^2^	Primary atom site location: structure-invariant direct methods
Least-squares matrix: full	Secondary atom site location: difference Fourier map
*R*[*F*^2^ > 2σ(*F*^2^)] = 0.047	Hydrogen site location: inferred from neighbouring sites
*wR*(*F*^2^) = 0.113	H-atom parameters constrained
*S* = 0.97	*w* = 1/[σ^2^(*F*_o_^2^) + (0.0576*P*)^2^] where *P* = (*F*_o_^2^ + 2*F*_c_^2^)/3
1477 reflections	(Δ/σ)_max_ = 0.001
183 parameters	Δρ_max_ = 0.23 e Å^−^^3^
1 restraint	Δρ_min_ = −0.14 e Å^−^^3^

### Special details

**Table:**

Geometry. All esds (except the esd in the dihedral angle between two l.s. planes) are estimated using the full covariance matrix. The cell esds are taken into account individually in the estimation of esds in distances, angles and torsion angles; correlations between esds in cell parameters are only used when they are defined by crystal symmetry. An approximate (isotropic) treatment of cell esds is used for estimating esds involving l.s. planes.
Refinement. Refinement of *F*^2^ against ALL reflections. The weighted *R*-factor wR and goodness of fit *S* are based on *F*^2^, conventional *R*-factors *R* are based on *F*, with *F* set to zero for negative *F*^2^. The threshold expression of *F*^2^ > σ(*F*^2^) is used only for calculating *R*-factors(gt) etc. and is not relevant to the choice of reflections for refinement. *R*-factors based on *F*^2^ are statistically about twice as large as those based on *F*, and *R*- factors based on ALL data will be even larger.

### Fractional atomic coordinates and isotropic or equivalent isotropic displacement parameters (Å^2^)

**Table:**

	*x*	*y*	*z*	*U*_iso_*/*U*_eq_	Occ. (<1)
C1	0.3705 (5)	0.8439 (3)	0.5517 (2)	0.0525 (9)	
C2	0.4237 (5)	0.7941 (4)	0.6359 (3)	0.0643 (10)	
C3	0.4381 (5)	0.8594 (4)	0.7158 (3)	0.0736 (12)	
H3	0.4738	0.8268	0.7707	0.088*	
C4	0.4018 (5)	0.9661 (4)	0.7144 (3)	0.0700 (11)	
H4	0.4127	1.0060	0.7685	0.084*	
C5	0.3467 (5)	1.0212 (3)	0.6328 (3)	0.0614 (10)	
C6	0.3091 (6)	1.1331 (4)	0.6324 (3)	0.0780 (12)	
H6	0.3201	1.1728	0.6866	0.094*	
C7	0.2573 (8)	1.1848 (4)	0.5552 (4)	0.0927 (14)	
H7	0.2337	1.2596	0.5562	0.111*	
C8	0.2390 (7)	1.1264 (4)	0.4742 (3)	0.0899 (14)	
H8	0.2024	1.1621	0.4209	0.108*	
C9	0.2744 (6)	1.0171 (3)	0.4721 (3)	0.0706 (11)	
H9	0.2614	0.9793	0.4171	0.085*	
C10	0.3302 (4)	0.9598 (3)	0.5510 (2)	0.0520 (8)	
C11	0.3571 (5)	0.7794 (3)	0.4722 (2)	0.0568 (9)	
H11	0.3241	0.8133	0.4175	0.068*	
C12	0.3841 (5)	0.6111 (3)	0.3898 (3)	0.0577 (9)	
C13	0.3368 (6)	0.5017 (3)	0.3975 (3)	0.0743 (10)	
C14	0.3288 (6)	0.4347 (4)	0.3228 (5)	0.0930 (15)	
H14	0.2959	0.3616	0.3296	0.112*	
C15	0.3693 (7)	0.4754 (5)	0.2380 (4)	0.0916 (16)	
H15	0.3627	0.4302	0.1868	0.110*	
C16	0.4199 (6)	0.5835 (4)	0.2282 (3)	0.0830 (13)	
H16	0.4484	0.6111	0.1704	0.100*	
C17	0.4283 (5)	0.6505 (3)	0.3039 (3)	0.0689 (11)	
H17	0.4642	0.7231	0.2971	0.083*	
F1	0.2925 (4)	0.4629 (2)	0.4811 (2)	0.1113 (10)	
N1	0.3887 (4)	0.6741 (2)	0.4711 (2)	0.0612 (8)	
H1B	0.4135	0.6418	0.5220	0.073*	0.43 (6)
O1	0.4613 (5)	0.6891 (2)	0.6408 (2)	0.0823 (9)	
H1A	0.4242	0.6584	0.5943	0.124*	0.57 (6)

### Atomic displacement parameters (Å^2^)

**Table:**

	*U*^11^	*U*^22^	*U*^33^	*U*^12^	*U*^13^	*U*^23^
C1	0.0475 (17)	0.062 (2)	0.048 (2)	−0.0027 (16)	−0.0010 (14)	−0.0002 (18)
C2	0.055 (2)	0.076 (3)	0.062 (3)	−0.003 (2)	0.0028 (18)	0.008 (2)
C3	0.068 (2)	0.106 (4)	0.047 (2)	0.005 (2)	0.0024 (19)	0.007 (2)
C4	0.062 (2)	0.099 (3)	0.049 (2)	−0.007 (2)	0.0014 (19)	−0.007 (2)
C5	0.055 (2)	0.072 (3)	0.057 (2)	−0.0125 (18)	0.0069 (17)	−0.010 (2)
C6	0.081 (3)	0.076 (3)	0.077 (3)	−0.012 (2)	0.015 (2)	−0.020 (3)
C7	0.118 (4)	0.057 (2)	0.103 (4)	−0.001 (3)	0.016 (3)	−0.004 (3)
C8	0.127 (4)	0.062 (3)	0.081 (3)	0.013 (3)	−0.003 (3)	0.006 (2)
C9	0.096 (3)	0.061 (2)	0.055 (2)	0.004 (2)	−0.003 (2)	0.0001 (18)
C10	0.0468 (18)	0.056 (2)	0.053 (2)	−0.0045 (15)	0.0006 (15)	0.0017 (18)
C11	0.055 (2)	0.057 (2)	0.059 (2)	0.0004 (17)	−0.0048 (18)	0.0079 (17)
C12	0.0473 (18)	0.052 (2)	0.073 (3)	0.0021 (16)	−0.0060 (18)	−0.006 (2)
C13	0.062 (2)	0.056 (3)	0.104 (2)	0.0001 (19)	−0.002 (2)	−0.005 (2)
C14	0.074 (3)	0.055 (3)	0.151 (5)	−0.001 (2)	−0.011 (3)	−0.010 (3)
C15	0.075 (3)	0.087 (4)	0.114 (4)	0.014 (3)	−0.007 (3)	−0.040 (3)
C16	0.075 (3)	0.094 (4)	0.080 (3)	0.014 (3)	0.000 (2)	−0.017 (3)
C17	0.067 (2)	0.070 (3)	0.070 (2)	0.007 (2)	0.001 (2)	−0.008 (2)
F1	0.124 (2)	0.0774 (17)	0.133 (2)	−0.0092 (16)	0.007 (2)	0.0304 (16)
N1	0.0596 (18)	0.057 (2)	0.067 (2)	0.0015 (15)	−0.0012 (15)	0.0019 (16)
O1	0.098 (2)	0.0781 (19)	0.0710 (19)	0.0091 (18)	0.0012 (16)	0.0213 (16)

### Geometric parameters (Å, °)

**Table:**

C1—C11	1.405 (5)	C9—H9	0.93
C1—C2	1.424 (5)	C11—N1	1.306 (4)
C1—C10	1.446 (5)	C11—H11	0.93
C2—O1	1.314 (5)	C12—C17	1.380 (5)
C2—C3	1.415 (5)	C12—C13	1.384 (5)
C3—C4	1.330 (6)	C12—N1	1.413 (4)
C3—H3	0.93	C13—F1	1.347 (5)
C4—C5	1.426 (6)	C13—C14	1.363 (6)
C4—H4	0.93	C14—C15	1.365 (7)
C5—C6	1.394 (6)	C14—H14	0.93
C5—C10	1.413 (5)	C15—C16	1.378 (7)
C6—C7	1.344 (6)	C15—H15	0.93
C6—H6	0.93	C16—C17	1.375 (6)
C7—C8	1.387 (6)	C16—H16	0.93
C7—H7	0.93	C17—H17	0.93
C8—C9	1.360 (5)	N1—H1B	0.86
C8—H8	0.93	O1—H1A	0.82
C9—C10	1.406 (5)		
			
C11—C1—C2	119.3 (3)	C9—C10—C1	123.5 (3)
C11—C1—C10	122.0 (3)	C5—C10—C1	119.7 (3)
C2—C1—C10	118.7 (3)	N1—C11—C1	123.4 (3)
O1—C2—C3	119.4 (4)	N1—C11—H11	118.3
O1—C2—C1	121.3 (4)	C1—C11—H11	118.3
C3—C2—C1	119.3 (4)	C17—C12—C13	117.9 (4)
C4—C3—C2	121.6 (4)	C17—C12—N1	124.4 (3)
C4—C3—H3	119.2	C13—C12—N1	117.7 (4)
C2—C3—H3	119.2	F1—C13—C14	120.0 (4)
C3—C4—C5	122.1 (4)	F1—C13—C12	118.2 (4)
C3—C4—H4	118.9	C14—C13—C12	121.8 (5)
C5—C4—H4	118.9	C13—C14—C15	119.7 (5)
C6—C5—C10	120.1 (4)	C13—C14—H14	120.2
C6—C5—C4	121.4 (4)	C15—C14—H14	120.2
C10—C5—C4	118.5 (4)	C14—C15—C16	120.1 (5)
C7—C6—C5	121.2 (4)	C14—C15—H15	120.0
C7—C6—H6	119.4	C16—C15—H15	120.0
C5—C6—H6	119.4	C17—C16—C15	119.9 (5)
C6—C7—C8	119.9 (4)	C17—C16—H16	120.0
C6—C7—H7	120.1	C15—C16—H16	120.0
C8—C7—H7	120.1	C16—C17—C12	120.7 (4)
C9—C8—C7	120.4 (4)	C16—C17—H17	119.7
C9—C8—H8	119.8	C12—C17—H17	119.7
C7—C8—H8	119.8	C11—N1—C12	122.9 (3)
C8—C9—C10	121.7 (4)	C11—N1—H1B	118.6
C8—C9—H9	119.2	C12—N1—H1B	118.6
C10—C9—H9	119.2	C2—O1—H1A	109.5
C9—C10—C5	116.7 (3)		
			
C11—C1—C2—O1	0.3 (5)	C11—C1—C10—C9	0.3 (5)
C10—C1—C2—O1	179.8 (3)	C2—C1—C10—C9	−179.2 (4)
C11—C1—C2—C3	179.7 (3)	C11—C1—C10—C5	−179.7 (3)
C10—C1—C2—C3	−0.9 (5)	C2—C1—C10—C5	0.8 (5)
O1—C2—C3—C4	179.9 (4)	C2—C1—C11—N1	1.0 (5)
C1—C2—C3—C4	0.5 (6)	C10—C1—C11—N1	−178.5 (3)
C2—C3—C4—C5	−0.1 (6)	C17—C12—C13—F1	−179.7 (4)
C3—C4—C5—C6	180.0 (4)	N1—C12—C13—F1	2.0 (5)
C3—C4—C5—C10	0.1 (5)	C17—C12—C13—C14	−1.7 (6)
C10—C5—C6—C7	−0.1 (6)	N1—C12—C13—C14	−180.0 (4)
C4—C5—C6—C7	180.0 (4)	F1—C13—C14—C15	178.3 (4)
C5—C6—C7—C8	0.5 (8)	C12—C13—C14—C15	0.4 (6)
C6—C7—C8—C9	−0.4 (8)	C13—C14—C15—C16	0.7 (7)
C7—C8—C9—C10	0.0 (8)	C14—C15—C16—C17	−0.5 (7)
C8—C9—C10—C5	0.4 (6)	C15—C16—C17—C12	−0.9 (7)
C8—C9—C10—C1	−179.5 (4)	C13—C12—C17—C16	1.9 (6)
C6—C5—C10—C9	−0.3 (5)	N1—C12—C17—C16	−179.9 (4)
C4—C5—C10—C9	179.6 (3)	C1—C11—N1—C12	−176.7 (3)
C6—C5—C10—C1	179.6 (3)	C17—C12—N1—C11	30.5 (5)
C4—C5—C10—C1	−0.5 (5)	C13—C12—N1—C11	−151.4 (3)

### Hydrogen-bond geometry (Å, °)

**Table:**

*D*—H···*A*	*D*—H	H···*A*	*D*···*A*	*D*—H···*A*
O1—H1A···N1	0.82	1.82	2.535 (4)	144
N1—H1B···O1	0.86	1.86	2.535 (4)	134

## References

[bb1] Allen, F. H., Kennard, O., Watson, D. G., Brammer, L., Orpen, A. G. & Taylor, R. (1987). *J. Chem. Soc. Perkin Trans. 2*, pp. S1–19.

[bb2] Farrugia, L. J. (1997). *J. Appl. Cryst.* **30**, 565.

[bb3] Farrugia, L. J. (1999). *J. Appl. Cryst.* **32**, 837–838.

[bb4] Hökelek, T., Kılıç, Z., Işıklan, M. & Toy, M. (2000). *J. Mol. Struct.* **523**, 61–69.

[bb5] Kaitner, B. & Pavlovic, G. (1996). *Acta Cryst.* C**52**, 2573–2575.

[bb6] Karabıyık, H., Güzel, B., Aygün, M., Boğa, G. & Büyükgüngör, O. (2007). *Acta Cryst.* C**63**, o215–o218. 10.1107/S0108270107005689 17413230

[bb7] Kargar, H., Jamshidvand, A., Fun, H.-K. & Kia, R. (2009). *Acta Cryst.* E**65**, m403–m404. 10.1107/S1600536809008721 PMC2968910 21582349

[bb8] Lozier, R. H., Bogomolni, R. A. & Stoeckenius, W. (1975). *Biophys. J.* **15**, 955–962. 10.1016/S0006-3495(75)85875-9 PMC1334761 1182271

[bb9] Nazır, H., Yıldız, M., Yılmaz, H., Tahir, M. N. & Ülkü, D. (2000). *J. Mol. Struct.* **524**, 241–250.

[bb10] Odabaşoğlu, M., Albayrak, Ç. & Büyükgüngör, O. (2005). *Acta Cryst.* E**61**, o425–o426. 10.1107/S0108270105005500 15805639

[bb11] Sheldrick, G. M. (2008). *Acta Cryst.* A**64**, 112–122. 10.1107/S0108767307043930 18156677

[bb12] Stoe & Cie (2002). *X-AREA* and *X-RED32* Stoe & Cie, Darmstadt, Germany.

[bb13] Tanak, H., Bingöl Alpaslan, Y., Yavuz, M., Ağar, E., Erşahin, F. & Büyükgüngör, O. (2009). *Acta Cryst.* E**65**, o1572. 10.1107/S1600536809021278 PMC2969358 21582851

[bb14] Yeap, C. S., Kia, R., Kargar, H. & Fun, H.-K. (2009). *Acta Cryst.* E**65**, m570–m571. 10.1107/S1600536809014500 PMC2977614 21583800

[bb15] Yıldız, M., Kılıç, Z. & Hökelek, T. (1998). *J. Mol. Struct.* **441**, 1–10.

